# Regulation of Monoclonal Antibody Immunotherapy by FcγRIIB

**DOI:** 10.1007/s10875-016-0247-8

**Published:** 2016-02-27

**Authors:** Richard J. Stopforth, Kirstie L. S. Cleary, Mark S. Cragg

**Affiliations:** Antibody & Vaccine Group, Cancer Sciences Unit, Faculty of Medicine, University of Southampton, Southampton General Hospital, Southampton, SO16 6YD UK

**Keywords:** Monoclonal antibody, immunotherapy, antigenic modulation, FcγRIIB, CD32B, immunomodulation

## Abstract

Monoclonal antibodies (mAb) are revolutionising the treatment of many different diseases. Given their differing mode of action compared to most conventional chemotherapeutics and small molecule inhibitors, they possess the potential to be independent of common modes of treatment resistance and can typically be combined readily with existing treatments without dose-limiting toxicity. However, treatments with mAb rarely result in cure and so a full understanding of how these reagents work and can be optimised is key for their subsequent improvement. Here we review how an understanding of the biology of the inhibitory Fc receptor, FcγRIIB (CD32B), is leading to the development of improved mAb treatments.

## Introduction

Fc gamma receptors (FcγRs) constitute a family of receptors for Immunoglobulin G (IgG) molecules. There are six FcγR family members in humans (FcγRI (CD64); FcγIIA (CD32A); FcγRIIB (CD32B); FcγRIIC (CD32C); FcγRIIIA (CD16A) and FcγRIIIB (CD16B)) and four in mice (FcγRI, FcγRIIB, FcγIII and FcγRIV), with known variations in their cellular expression patterns [1] and affinities for monomeric IgG [2]. CD32B is the only inhibitory member of the FcγR family in mice and humans, and binds to monomeric IgG molecules with a low affinity [2]. It is expressed by B cells and myeloid cells at variable levels depending on the cell subtype and activation state [1]. In addition, CD32B is expressed on the surface of B cell leukaemia/lymphoma cells [3]. Two isoforms of CD32B exist in mouse and man, CD32B1 and CD32B2 [4, 5]; differences in the cytoplasmic domain of the latter results in a greater propensity to internalise [4].

Monoclonal antibodies (mAbs) represent an established treatment paradigm for a number of diseases including cancer, and either aim to induce target cell destruction or immune cell activation, such as in the case of anti-CD20 [6] or anti-CD40 [7, 8] mAbs, respectively. Following binding to their cognate antigens, mAbs may engage Fc gamma receptors (FcγR) on the surface of immune cells. To this end, the use of FcγR knockout mice [9, 10], and more recently human FcγR transgenic [11] or “humanised” mice [12] has provided overwhelming evidence for Fc-FcγR engagement being central to the mechanism of action of most mAb therapies. Although less direct evidence is available in humans, FcγR polymorphisms associated with greater affinities for the Fc region of IgG have been associated with favourable therapeutic responses and autoimmune disease susceptibility in some cases (reviewed in [13]).

Intriguingly, it has been shown that engagement of the inhibitory CD32B may be either detrimental [3, 9] or beneficial [7, 8] to therapeutic efficacy depending on the type of mAb and context, as will be discussed below.

## CD32B and Regulation of Immune Responses

The physiological function of CD32B is to regulate the activation state of the expressing cell through interaction with other cell surface receptors. In B cells, co-ligation of the B cell receptor (BCR) with CD32B on the same cell (in *cis*) via antibody-coated antigen (immune complex), provides a mechanism to limit activation, proliferation and/or antibody secretion [14]. Moreover, CD32B has been shown to provide a threshold for activation of cells co-expressing activatory FcγRs and CD32B [15]. For example, CD32B^−/−^ macrophages produced more robust calcium (Ca^2+^) flux following surface ligation of activatory FcγR and mediated greater phagocytosis in comparison to wild type macrophages.

The nature of CD32B inhibitory signalling has recently been reviewed elsewhere [16]. Briefly, early work showed that a 13 amino acid sequence in the cytoplasmic domain of CD32B, containing what is now known as an immunoreceptor tyrosine-based inhibitory motif (ITIM), was responsible for the inhibition of Ca^2+^ flux following BCR-CD32B co-ligation in B cells [17]. Later work identified that the recruitment of the SH2-domain containing 5′-inositol phosphatase SHIP-1 to this motif is required [18]. SHIP-1 is able to down-regulate activatory receptor signalling in several ways but central to its function is the dephosphorylation of membrane phosphatidyl inositol-3,4,5-trisphosphate (PIP_3_) to phosphatidyl inositol-3,4-bisphosphate (PIP_2_); preventing the recruitment of plekstrin homology (PH)-domain containing signalling proteins such as Btk to the cell membrane [19].

However, it is becoming clear that the intracellular, ITIM-containing domain of CD32B is not always required for its inhibitory functions. One example is the intracellular region-independent, transmembrane region-dependent clustering of the BCR and CD19 following BCR-CD32B co-ligation, which was observed in imaging studies [20]. Further still, there is evidence that CD32B is not the only FcγR capable of mediating inhibitory signalling, with inhibitory ITAM signalling (ITAMi) being observed downstream of activatory FcγR ligation in some circumstances (reviewed in [16]).

The role of CD32B in preventing excessive immune responses resulting in autoimmune disease has recently been reviewed elsewhere [21]. However, a CD32B polymorphism associated with autoimmune disease that results in an I232T amino acid change in the transmembrane region of CD32B is noteworthy as it has provided insight into the biology of CD32B [22, 23]. Functionally, this polymorphism has been linked to reduced CD32B function and hence greater ‘activatory’ signalling. In B cells, 232 T CD32B was less able to inhibit Ca^2+^ flux and downstream signalling following BCR-CD32B crosslinking [23]. This was also observed in transfected macrophage cell lines following activatory FcγR (CD64) crosslinking, and monocyte derived macrophages from 232 T donors had a more activated phenotype and more readily phagocytosed antibody-opsonised bacteria [22]. The mechanism responsible for this has been shown to be reduced localisation of CD32B 232 T into lipid rafts [22, 23]. Intriguingly, studies have suggested that this polymorphism, despite being associated with an increased incidence of systemic lupus erythematosus, may provide a selective advantage in areas affected by malaria [24].

**Fig. 1:**
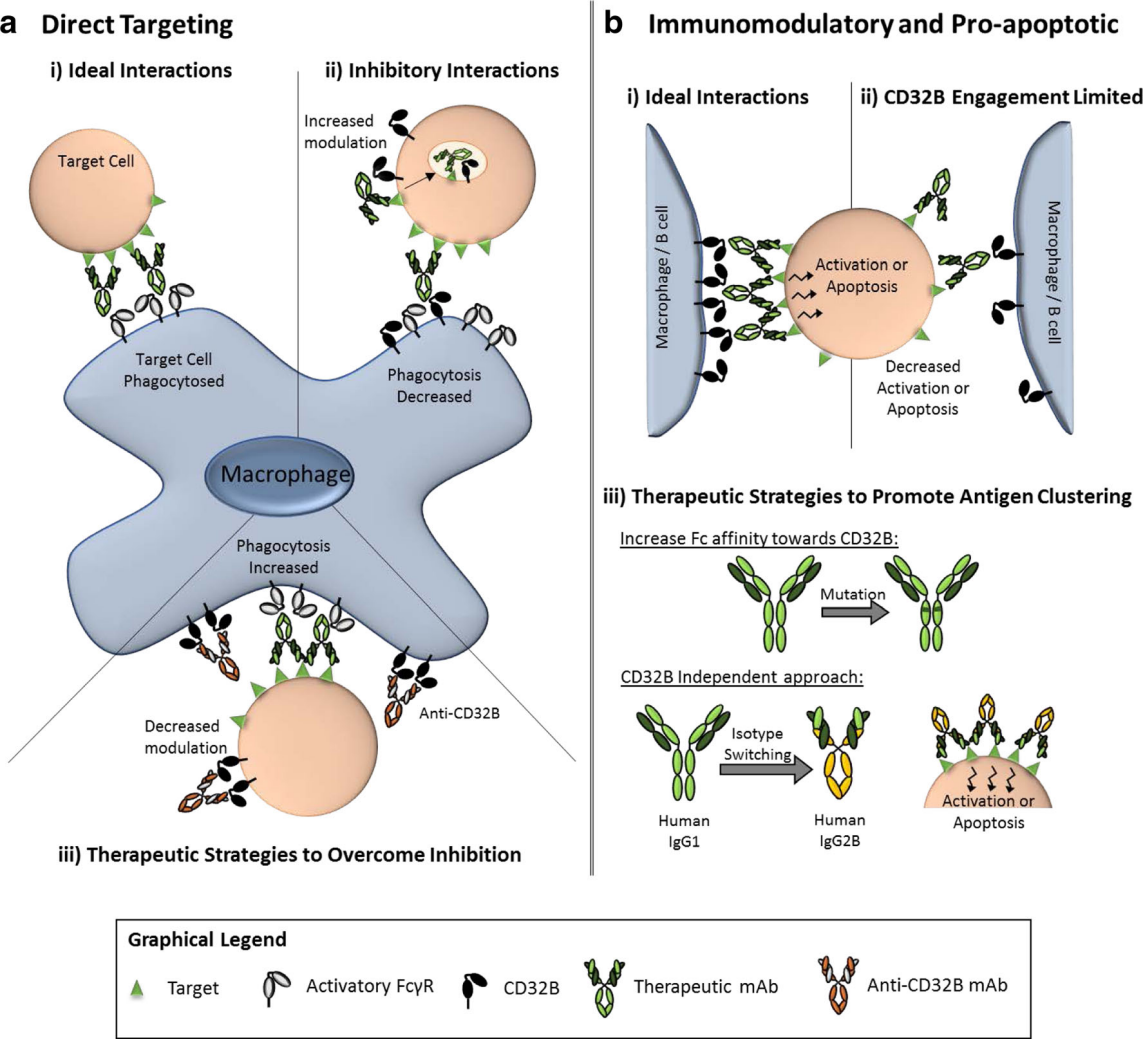
Means through which CD32B modulates mAb immunotherapy and how it may be overcome. **a** Direct targeting mAb. Clearance of tumour cells opsonised with anti-tumour mAbs requires interaction with activatory FcγR on the surface of macrophages, resulting in downstream phagocytosis of the tumour cell (*i*). The inhibitory FcγR CD32B may inhibit this process, either by promoting target antigen: mAb complex internalisation (modulation) from the target cell surface, and/or by inhibiting activatory FcγRs on macrophages (*ii*). One strategy to overcome this inhibition of target cell phagocytosis may be to target CD32B on the target and/or effector cell with anti-CD32B mAbs (*iii*). **b** Immunomodulatory and pro-apoptotic mAb. In contrast to (**a**), mAbs which aim to induce immune cell activation (immunomodulatory mAbs) or target cell apoptosis (pro-apoptotic mAbs) have been shown to benefit from interactions with the inhibitory FcγR CD32B (*i*). Interaction with CD32B may be limited by a low affinity for CD32B or the availability of CD32B+ cells (*ii*). One approach to overcome this may be to introduce mutations (S267E/L328F) into the constant region of these antibodies to increase CD32B binding (*iii*, *top*). Alternatively, the human IgG2B isotype has been shown to have greater agonisitic properties by inducing greater target antigen clustering, independently of CD32B binding (*iii*, *bottom*)

In addition to effects on immune responses and propensity to disease, CD32B is able to regulate IgG-based mAb therapeutics in several ways (Fig. 1).

### CD32B-Mediated Regulation of Monoclonal Antibody Efficacy

The seminal study performed by Clynes et al. demonstrated conclusively that FcγR regulate the activity of so-called direct-targeting mAb in the setting of tumour therapy. These authors showed the absence of therapeutic responses in activatory FcγR-deficient (γ chain −/−) mice and conversely an improvement in mice lacking CD32B [9]. Given this improvement in CD32^−/−^ mice and the fact that FcγRs other than CD16A cannot normally be detected on the surface of natural killer (NK) cells [1] these observations have been used as evidence against a role for NK cells in the mechanism of action of therapeutic mAbs. As macrophages co-express both activatory FcγRs and CD32B [15], these studies indicate that loss of CD32B may increase effector function (phagocytosis) *in vivo*, as has been shown *in vitro* [15], and therefore a role for these effectors in the mechanism of action of therapeutic mAbs. Notably, the anti-tumour potential of tumour-targeting mAbs has been shown to be abolished in mice depleted of macrophages and other phagocytes [25]. Moreover, convincing intravital microscopy studies have also recently provided direct evidence for a role of macrophages *in vivo* [26, 27]. Therefore, despite evidence from human peripheral blood mononuclear cell (PBMC) assays indicating the importance of NK cells following mAb therapy in some settings, the growing consensus in the field is that macrophages may have a more prominent role in the mechanism of action of therapeutic mAbs than first thought.

IgG antibody isotypes have varying affinities for activatory and inhibitory FcγR. Specifically, mouse IgG1 binds FcγRIII and FcγRII whereas mouse IgG2a antibodies bind only weakly to FcγRII but strongly to FcγRI and IV [10]. A ratio of relative activatory to inhibitory FcγR binding ability can therefore be calculated, known as the ‘activatory: inhibitory’ (A:I) ratio, and used to predict therapeutic efficacy [10]. Notably, in mouse models, mouse IgG2a mAbs (high A:I) are effective in clearing tumour cells and platelets in melanoma and idiopathic thrombocytopenic purpura models, respectively, whereas mouse IgG1 mAbs (low A:I) result in poorer depletion [10]. In humans, the distinction is less clear, although human IgG1 and 3 are perhaps most equivalent to mouse IgG2a, and able to deplete platelets in humanized mice expressing human FcγRs more effectively than human IgG2 and 4 isotypes [12].

Finally, the ability of human IgG1 antibodies, which are capable of binding to macrophage FcγRIV but not NK cell FcγRIII, to deplete tumour cells in mouse models [28] provides further evidence for a role of macrophages over NK cells *in vivo*, at least in mice.

In addition to these observations, we recently observed a hitherto unappreciated facet of CD32B biology, relating to B cell targets.

### Antigenic Modulation of CD20: IgG Complexes

Work in our laboratory previously identified that certain direct-targeting mAb such as the type I anti-CD20 mAb Rituximab internalise from the surface of both malignant and autoimmune B cells in a process termed antigenic modulation, providing a potential resistance mechanism in the treatment of these diseases [25].

To our surprise, further work showed that CD32B was involved in this process [3], with modulation shown to correlate with the level of CD32B expression on the surface of the lymphoma or B cell [3]. Furthermore, our results suggested that *cis* interaction between the Fc portion of anti-CD20 mAb and CD32B on the same cell was responsible for internalisation, in a process termed antibody bipolar bridging. Although Rituximab leads to phosphorylation of CD32B in *cis* as a result of this process, it was shown that the intracellular domain of CD32B was not required for modulation [29].

Nevertheless, such antigenic modulation is of therapeutic relevance for several reasons. Firstly, modulation of CD20 by type I anti-CD20 mAbs was shown to limit tumour cell killing by mouse and human immune effector cells [28]. Secondly, the decline in effector cell mediated killing was not observed in the context of type II anti-CD20 mAbs, consistent with the lack of modulation [28]. Moreover, type II anti-CD20 mAbs led to greater B cell depletion *in vivo* in comparison to type I anti-CD20 mAbs [28] and were more effective in a clinical trial involving chronic lymphocytic leukaemia (CLL) patients [6]. Thirdly, considering the link between CD32B expression and antigenic modulation, the expression level of CD32B on B cell leukaemias/lymphomas may be able to predict response to mAb therapy. To this end, it was shown that mantle cell lymphoma patients with greater tumour CD32B expression had a shorter progression-free survival following Rituximab containing immunochemotherapy [3]. Similar observations were made in the setting of Rituximab monotherapy in follicular lymphoma [30]. Finally, the modulation of type I anti-CD20 mAbs from the cell surface may contribute to a reduction in antibody half-life [28].

### Contrasting Effect of CD32B with Immune-Modulatory and Pro-Apoptotic Antibodies

Unexpectedly, *in vitro* and *in vivo* experiments performed by several independent research groups showed that the ability of immune modulatory mAbs targeting the tumour necrosis factor receptor (TNFR) superfamily member CD40 required binding to CD32B for optimal function [7, 8, 31]. These ‘agonistic’ mAbs, in contrast to Rituximab in the scenario described above, engage CD32B on a different cell than the target (*in trans*) to elicit antigen presenting cell (APC) activation and subsequent adaptive immune responses [31]. As a result, these reagents achieve most potent agonism as mouse IgG1 antibodies due to preferential CD32B binding, in contrast to Rituximab which works optimally as a mouse IgG2a in mice (see above). Similarly, pro-apoptotic mAbs targeting death receptors such as DR-5 have shown a requirement for CD32B binding in mouse models [32, 33]. These findings therefore provide a model whereby CD32B can promote target antigen clustering in *trans* leading to target cell activation or apoptotic signalling. However, similar to CD20:mAb antigenic modulation, the intracellular domain of CD32B is not required for these clustering functions [7, 34].

### Side-Effects Downstream of CD32B Engagement

Although CD32B binding may promote immune-modulatory or pro-apoptotic mAb activity, a limitation may be an increased risk for side effects. Notably, a pro-apoptotic, anti-Fas mAb caused CD32B-dependent lethal hepatotoxicity in mice [34]. This was proposed to be due to sinusoidal endothelial cell CD32B expression. Similarly, the anti-DR-5 mAb MD5-1 induced CD32B-dependent hepatotoxicity at high doses in mice [33].

In a different context, the administration of an anti-CD28 human IgG4 mAb led to the hospitalisation of 6 clinical trial participants, and their treatment for cytokine release syndrome [35]. Studies with PBMCs have since shown that FcγRs, and in particular CD32B, can provide a crosslinking function (in *trans*) that is required to elicit potent cytokine release from TGN1412-opsonised T lymphocytes [36]. Furthermore, in these assays, a high density pre-culture is required which results in a large upregulation of CD32B on the monocytes [37]. It is these CD32B^high^ monocytes, (as well as CD32B^+^ B cells in sufficient number), that are capable of clustering the Fc region of TGN1412 bound to target CD28 antibodies on the surface of T cells and promoting inflammatory cytokine release. The promotion of target antigen clustering by CD32B has therefore been proposed as a potential mechanism to explain the severe side effects observed in this trial.

Although details of the potential requirement for CD32B in this setting *in vivo* are still being elucidated, these studies highlight an underappreciated role of human IgG molecules, including IgG4, to bind to CD32B when present as an immune complex, as has been previously reported [38]. This emphasises the need to consider alternative assays other than the assessment of monomeric IgG-FcγR interactions via surface plasmon resonance in the screening of candidate therapeutic mAbs.

## Therapeutic Strategies

As discussed above, CD32B may provide beneficial or detrimental contributions to the efficacy of therapeutic mAbs depending on the context. Treatments are therefore being developed to either abrogate the inhibitory functions of CD32B, harness the ability of CD32B to promote mAb: target clustering and downstream signalling, or overcome the requirement for CD32B binding (Fig. 1).

### Anti-CD32B Antibodies

One approach to inhibit CD32B function is to develop mAbs targeting this receptor. Generation of both anti-mouse [39] and anti-human [40, 41] CD32B-specific mAbs has been achieved, the latter, despite the close similarity between the extracellular domains of the activatory FcγR CD32A and CD32B.

Anti-mouse CD32B mAbs were identified that induced or prevented the phosphorylation of the CD32B cytoplasmic region, so called agonistic or antagonistic mAbs, respectively [39]. Despite mediating direct cell death [39] and antibody-dependent cell-mediated phagocytosis (ADCP) [42], studies of anti-CD32B antibodies in mice were thwarted by rapid internalisation and loss of antibodies from the circulation [42], similar to anti-CD20 modulation (see above), although this was not applicable to the human system.

Anti-human CD32B mAbs were also generated and shown to be either agonistic or antagonistic with regards CD32B phosphorylation [41]. A candidate antagonistic mAb (6G11) was taken forward for further investigation. Similar to previous studies of anti-human CD32B [40], this study confirmed the ability of anti-CD32B mAbs to mediate direct cell death, antibody-dependent cell-mediated cytotoxicity (ADCC) and ADCP *in vitro*. Anti-CD32B N297Q mAbs, which cannot interact with FcγRs through their Fc, were previously shown not to induce ADCC or ADCP *in vitro* [40]. However, CD32B-specific N297Q mAbs were capable of reducing antigenic modulation induced by Rituximab (see above), which corresponded to an increase in effector-mediated killing [41]. This suggests that anti-CD32B mAbs may also function in an Fc-independent manner to inhibit CD20:mAb modulation and maintain anti-CD20:mAb Fc region recognition by effector cells. Inhibition of CD32B co-ligation with activatory FcγR on immune cells is another possible Fc-independent function.

This study, in comparison to previous studies of anti-human CD32B [40], used human CD32B transgenic mice that lack the endogenous mouse receptor [41]. Key findings include the ability of N297Q mAbs to enhance B cell depletion only when combined with Rituximab *in vivo*, and the greater tumour depletion observed in mice xenografted with primary CLL cells when treated with a Rituximab plus anti-CD32B mAb in combination.

Considering the expression of CD32B on both the surface of B cell leukaemia/lymphoma cells and immune effector cells, it is at present difficult to distinguish the relative contribution of inhibition of tumour cell antigenic modulation versus blocking of inhibitory CD32B signalling in immune cells to the therapeutic effects. Our current studies aim to dissect these various aspects.

### Antibody Fc Region Engineering

One avenue to increase the efficacy of therapeutic mAbs has been to engineer their Fc regions to have altered affinities for FcγRs; typically higher and lower affinities to activatory and inhibitory FcγRs, respectively. This may be achieved by mutagenesis [43] or by altering the glycan groups attached to residue N297 in the Fc region of antibodies [44]. Recently, specific mutations of the mAb Fc backbone which selectively increase binding affinities to individual human FcγRs have been linked to distinct effector mechanisms [11], contributing to our understanding of mAb mechanisms of action.

Antibody variants, such as S239D/I332E/A330L, may be selected which have improved binding to activatory over inhibitory FcγRs, and hence activatory: inhibitory ratios [43], which may reduce the inhibition of effector cell signalling by CD32B. In contrast, mutations have also been considered to increase mAb Fc region binding to CD32B, such as in the context of autoimmune disease treatment [45]. One particular polymorphism (S267E/L328F or SE/LF) is known to increase the Fc region binding affinity of human IgG1 to human CD32B by 430-fold, and binding to CD32B-transfected cells by 310-fold [45]. Consequently, the SE/LF mutant on an anti-CD19 backbone was able to limit B cell activation downstream of CD79b stimulation. Although this approach would likely dampen the efficacy of tumour-targeting mAbs which require activatory FcγR binding as opposed to CD32B for function, mutating these residues has been shown to augment the efficacy of mAbs targeting TNFR superfamily members which benefit from CD32B engagement (see above). Specifically, the treatment of human CD32B transgenic mice with human IgG1 anti-DR5 antibodies with the S267E mutation resulted in greater tumour regression and/or survival in comparison to unmodified mAb, which corresponded with enhanced tumour cell apoptosis *in vitro* [33]. Similarly, an anti-CD40 S267E/L328F human IgG1 mAb induced greater B cell activation and proliferation *in vitro* [31]. Moreover, human IgG1 anti-CD40 S267E was more agonistic in human CD32B-transgenic mice, with favourable tumour depletion and prolonged survival [8].

However, strategies to increase the affinity of the Fc portion of mAbs for CD32B may be limited by an increased potential for side-effects (see above). In particular, despite having favourable efficacy, the S267E version of a human IgG1 anti-DR5 mAb resulted in a significant increase in liver enzyme release [33].

Afucosylation, (removal of the fucose groups attached to the N297 glycan of antibodies), is known to increase Fc binding to the activatory FcγR CD16A and consequently enhance effector-mediated killing (ADCC) [44]. Although the glycomodified (afucosylated) type II anti-CD20 mAb obinutuzumab combined with chlorambucil chemotherapy was more efficacious in comparison with rituximab plus chemotherapy in clinical trials [6], results from *in vitro* and *in vivo* studies of a non-glycomodified version of obinutuzumab suggest that the superiority of type II anti-CD20 mAbs is most likely a result of their type II nature rather than glycomodification [28]. For example, it should be considered that the relatively less antigenic modulation mediated by type II anti-CD20 mAbs may be responsible for the differences in efficacy observed in patients.

### CD32B-Independent mAb: Antigen Clustering

Although agonistic anti-CD40 mAbs required CD32B binding in mice (see above), the affinity of human IgG1 to CD32B is low, at least in monomeric form [2], and enhancing CD32B binding may increase hepatotoxicity [34]. Therefore, designing agonistic mAbs that avoid or overcome the requirement for CD32B binding may be desirable. Intriguingly, the human IgG2 isoform IgG2B has recently been shown to cluster target antigens and promote cell signalling and activation independently of CD32B binding [46]. Considering the fact that monomeric IgG2 has the lowest affinity for CD32B out of all human IgG isotypes [2], the use of this isotype may also provide an opportunity to limit the side effects observed with agonistic antibodies that bind to CD32B in mice (see above).

### Future Directions

The beneficial and detrimental influences of CD32B on mAb therapeutic efficacy have been discussed, as well as potential therapeutic strategies to take advantage of CD32B binding, or inhibit CD32B function. The pre-clinical development of anti-CD32B mAbs has provided the background for a clinical trial of a lead candidate (BI-1206) due to begin later this year. However, unanswered questions include: why targeting CD32B with different mAbs leads to different biological outcomes (CD32B phosphorylation or not), and specifically how structural changes or clustering in the cell membrane following antibody: CD32B interactions result in these differences.

Finally, although this review has largely focussed on the context of B cell targets, there is also evidence that CD32B is expressed by other target cells (e.g. metastatic melanoma cells), and a mechanism whereby CD32B inhibits ADCC of these cells has also been proposed [47] providing the potential for therapeutic blocking of CD32B in this setting. Finally, it remains a possibility that blocking CD32B on effector cells may be useful in additional settings where other deleting mAbs are being used but where CD32B is not expressed on the target cell.

## References

[1] Tutt AL, James S, Laversin SA, Tipton TR, Ashton-Key M, French RR (2015). Development and characterization of monoclonal antibodies specific for mouse and human Fcgamma receptors. J Immunol.

[2] Bruhns P, Iannascoli B, England P, Mancardi DA, Fernandez N, Jorieux S (2009). Specificity and affinity of human Fcgamma receptors and their polymorphic variants for human IgG subclasses. Blood.

[3] Lim SH, Vaughan AT, Ashton-Key M, Williams EL, Dixon SV, Chan HT (2011). Fc gamma receptor IIb on target B cells promotes rituximab internalization and reduces clinical efficacy. Blood.

[4] Budde P, Bewarder N, Weinrich V, Schulzeck O, Frey J (1994). Tyrosine-containing sequence motifs of the human immunoglobulin G receptors FcRIIb1 and FcRIIb2 essential for endocytosis and regulation of calcium flux in B cells. J Biol Chem.

[5] Brooks DG, Qiu WQ, Luster AD, Ravetch JV (1989). Structure and expression of human IgG FcRII(CD32). Functional heterogeneity is encoded by the alternatively spliced products of multiple genes. J Exp Med.

[6] Goede V, Fischer K, Busch R, Engelke A, Eichhorst B, Wendtner CM (2014). Obinutuzumab plus chlorambucil in patients with CLL and coexisting conditions. N Engl J Med.

[7] White AL, Chan HT, Roghanian A, French RR, Mockridge CI, Tutt AL (2011). Interaction with FcgammaRIIB is critical for the agonistic activity of anti-CD40 monoclonal antibody. J Immunol.

[8] Li F, Ravetch JV (2011). Inhibitory Fcgamma receptor engagement drives adjuvant and anti-tumor activities of agonistic CD40 antibodies. Science.

[9] Clynes RA, Towers TL, Presta LG, Ravetch JV (2000). Inhibitory Fc receptors modulate in vivo cytotoxicity against tumor targets. Nat Med.

[10] Nimmerjahn F, Ravetch JV (2005). Divergent immunoglobulin g subclass activity through selective Fc receptor binding. Science.

[11] DiLillo DJ, Ravetch JV. Differential Fc-Receptor engagement drives an anti-tumor vaccinal effect. Cell. 2015;161(5):1035–45. 10.1016/j.cell.2015.04.016 PMC4441863 25976835

[12] Schwab I, Lux A, Nimmerjahn F. Pathways responsible for human autoantibody and therapeutic intravenous IgG Activity in humanized mice. Cell Rep. 2015;13(3):610–20. 10.1016/j.celrep.2015.09.013 26456831

[13] Hargreaves CE, Rose-Zerilli MJ, Machado LR, Iriyama C, Hollox EJ, Cragg MS (2015). Fcgamma receptors: genetic variation, function, and disease. Immunol Rev.

[14] Phillips NE, Parker DC (1983). Fc-dependent inhibition of mouse B cell activation by whole anti-mu antibodies. J Immunol.

[15] Clynes R, Maizes JS, Guinamard R, Ono M, Takai T, Ravetch JV (1999). Modulation of immune complex-induced inflammation in vivo by the coordinate expression of activation and inhibitory Fc receptors. J Exp Med.

[16] Getahun A, Cambier JC (2015). Of ITIMs, ITAMs, and ITAMis: revisiting immunoglobulin Fc receptor signaling. Immunol Rev.

[17] Muta T, Kurosaki T, Misulovin Z, Sanchez M, Nussenzweig MC, Ravetch JV (1994). A 13-amino-acid motif in the cytoplasmic domain of Fc gamma RIIB modulates B-cell receptor signalling. Nature.

[18] Ono M, Okada H, Bolland S, Yanagi S, Kurosaki T, Ravetch JV (1997). Deletion of SHIP or SHP-1 reveals two distinct pathways for inhibitory signaling. Cell.

[19] Bolland S, Pearse RN, Kurosaki T, Ravetch JV (1998). SHIP modulates immune receptor responses by regulating membrane association of Btk. Immunity.

[20] Xu L, Li G, Wang J, Fan Y, Wan Z, Zhang S (2014). Through an ITIM-independent mechanism the FcgammaRIIB blocks B cell activation by disrupting the colocalized microclustering of the B cell receptor and CD19. J Immunol.

[21] Espeli M, Smith KG, Clatworthy MR (2016). FcgammaRIIB and autoimmunity. Immunol Rev.

[22] Floto RA, Clatworthy MR, Heilbronn KR, Rosner DR, MacAry PA, Rankin A (2005). Loss of function of a lupus-associated FcgammaRIIb polymorphism through exclusion from lipid rafts. Nat Med.

[23] Kono H, Kyogoku C, Suzuki T, Tsuchiya N, Honda H, Yamamoto K (2005). FcgammaRIIB Ile232Thr transmembrane polymorphism associated with human systemic lupus erythematosus decreases affinity to lipid rafts and attenuates inhibitory effects on B cell receptor signaling. Hum Mol Genet.

[24] Willcocks LC, Carr EJ, Niederer HA, Rayner TF, Williams TN, Yang W (2010). A defunctioning polymorphism in FCGR2B is associated with protection against malaria but susceptibility to systemic lupus erythematosus. Proc Natl Acad Sci U S A.

[25] Beers SA, French RR, Chan HT, Lim SH, Jarrett TC, Vidal RM (2010). Antigenic modulation limits the efficacy of anti-CD20 antibodies: implications for antibody selection. Blood.

[26] Montalvao F, Garcia Z, Celli S, Breart B, Deguine J, Van Rooijen N, et al. The mechanism of anti-CD20-mediated B cell depletion revealed by intravital imaging. J Clin Invest. 2013;123(12):5098–103. 10.1172/JCI70972 PMC3859399 24177426

[27] Gul N, Babes L, Siegmund K, Korthouwer R, Bogels M, Braster R (2014). Macrophages eliminate circulating tumor cells after monoclonal antibody therapy. J Clin Invest.

[28] Tipton TR, Roghanian A, Oldham RJ, Carter MJ, Cox KL, Mockridge CI (2015). Antigenic modulation limits the effector cell mechanisms employed by type I anti-CD20 monoclonal antibodies. Blood.

[29] Vaughan AT, Chan CH, Klein C, Glennie MJ, Beers SA, Cragg MS (2015). Activatory and inhibitory Fcgamma receptors augment rituximab-mediated internalization of CD20 independent of signaling via the cytoplasmic domain. J Biol Chem.

[30] Lee CS, Ashton-Key M, Cogliatti S, Rondeau S, Schmitz SF, Ghielmini M, et al. Expression of the inhibitory Fc gamma receptor IIB (FCGR2B, CD32B) on follicular lymphoma cells lowers the response rate to rituximab monotherapy (SAKK 35/98). Br J Haematol. 2015;168(1):145–8. 10.1111/bjh.13071 25142001

[31] White AL, Chan HT, French RR, Beers SA, Cragg MS, Johnson PW (2013). FcgammaRIIB controls the potency of agonistic anti-TNFR mAbs. Cancer Immunol Immunother.

[32] Wilson NS, Yang B, Yang A, Loeser S, Marsters S, Lawrence D (2011). An Fcgamma receptor-dependent mechanism drives antibody-mediated target-receptor signaling in cancer cells. Cancer Cell.

[33] Li F, Ravetch JV (2012). Apoptotic and antitumor activity of death receptor antibodies require inhibitory Fcgamma receptor engagement. Proc Natl Acad Sci U S A.

[34] Xu Y, Szalai AJ, Zhou T, Zinn KR, Chaudhuri TR, Li X (2003). Fc gamma Rs modulate cytotoxicity of anti-Fas antibodies: implications for agonistic antibody-based therapeutics. J Immunol.

[35] Suntharalingam G, Perry MR, Ward S, Brett SJ, Castello-Cortes A, Brunner MD (2006). Cytokine storm in a phase 1 trial of the anti-CD28 monoclonal antibody TGN1412. N Engl J Med.

[36] Bartholomaeus P, Semmler LY, Bukur T, Boisguerin V, Romer PS, Tabares P (2014). Cell contact-dependent priming and Fc interaction with CD32+ immune cells contribute to the TGN1412-triggered cytokine response. J Immunol.

[37] Hussain K, Hargreaves CE, Roghanian A, Oldham RJ, Chan HT, Mockridge CI, et al. Upregulation of FcgammaRIIb on monocytes is necessary to promote the superagonist activity of TGN1412. Blood. 2015;125(1):102–10. 10.1182/blood-2014-08-593061 25395427

[38] Lux A, Yu X, Scanlan CN, Nimmerjahn F (2013). Impact of immune complex size and glycosylation on IgG binding to human FcgammaRs. J Immunol.

[39] Williams EL, Tutt AL, French RR, Chan HT, Lau B, Penfold CA (2012). Development and characterisation of monoclonal antibodies specific for the murine inhibitory FcgammaRIIB (CD32B). Eur J Immunol.

[40] Rankin CT, Veri MC, Gorlatov S, Tuaillon N, Burke S, Huang L (2006). CD32B, the human inhibitory Fc-gamma receptor IIB, as a target for monoclonal antibody therapy of B-cell lymphoma. Blood.

[41] Roghanian A, Teige I, Martensson L, Cox KL, Kovacek M, Ljungars A (2015). Antagonistic human FcgammaRIIB (CD32B) antibodies have anti-tumor activity and overcome resistance to antibody therapy in vivo. Cancer Cell.

[42] Williams EL, Tutt AL, Beers SA, French RR, Chan CH, Cox KL (2013). Immunotherapy targeting inhibitory fcgamma receptor IIB (CD32b) in the mouse is limited by monoclonal antibody consumption and receptor internalization. J Immunol.

[43] Lazar GA, Dang W, Karki S, Vafa O, Peng JS, Hyun L (2006). Engineered antibody Fc variants with enhanced effector function. Proc Natl Acad Sci U S A.

[44] Shields RL, Lai J, Keck R, O’Connell LY, Hong K, Meng YG (2002). Lack of fucose on human IgG1 N-linked oligosaccharide improves binding to human Fcgamma RIII and antibody-dependent cellular toxicity. J Biol Chem.

[45] Chu SY, Vostiar I, Karki S, Moore GL, Lazar GA, Pong E (2008). Inhibition of B cell receptor-mediated activation of primary human B cells by coengagement of CD19 and FcgammaRIIb with Fc-engineered antibodies. Mol Immunol.

[46] White AL, Chan HT, French RR, Willoughby J, Mockridge CI, Roghanian A, et al. Conformation of the human immunoglobulin G2 hinge imparts superagonistic properties to immunostimulatory anticancer antibodies. Cancer Cell. 2015;27(1):138–48. 10.1016/j.ccell.2014.11.001 PMC4297290 25500122

[47] Cohen-Solal JF, Cassard L, Fournier EM, Loncar SM, Fridman WH, Sautes-Fridman C (2010). Metastatic melanomas express inhibitory low affinity fc gamma receptor and escape humoral immunity. Dermatol Res Pract.

